# Human virome in nasopharynx and tracheal secretion samples

**DOI:** 10.1590/0074-02760190198

**Published:** 2019-10-03

**Authors:** Larissa da Costa Souza, Rosana Blawid, João Marcos Fagundes Silva, Tatsuya Nagata

**Affiliations:** 1 Universidade de Brasília, Departamento de Biologia Celular, Pós-Graduação em Biologia Microbiana, Brasília, DF, Brasil; 2 Universidade Federal Rural de Pernambuco, Departamento de Agronomia, Recife, PE, Brasil; 3 Laboratório Central de Saúde Pública do Distrito Federal, Brasília, DF, Brasil; 4 Universidade de Brasília, Departamento de Biologia Celular, Pós-Graduação em Biologia Molecular, Brasília, DF, Brasil

**Keywords:** respiratory virus, public health, high-throughput sequencing, RT-qPCR

## Abstract

**BACKGROUND:**

In Brazil the implementation of the Sentinel Surveillance System of Influenza began in 2000. Central public health laboratories use reverse transcription-quantitative polymerase chain reaction (RT-qPCR) for diagnosis of respiratory viruses, but this protocol identifies only specific targets, resulted in inconclusive diagnosis for many samples. Thus, high-throughput sequencing (HTS) would be complementary method in the identification of pathogens in inconclusive samples for RT-qPCR or other specific detection protocols.

**OBJECTIVES:**

This study aimed to detect unidentified viruses using HTS approach in negative samples of nasopharynx/tracheal secretions by the standard RT-qPCR collected in the Federal District, Brazil.

**METHODS:**

Nucleic acids were extracted from samples collected in winter period of 2016 and subjected to HTS. The results were confirmed by the multiplex PR21 RT-qPCR, which identifies 21 respiratory pathogens.

**FINDINGS:**

The main viruses identified by HTS were of families *Herpesviridae*, *Coronaviridae*, *Parvoviridae* and *Picornaviridae*, with the emphasis on rhinoviruses. The presence of respiratory viruses in the samples was confirmed by the PR21 multiplex RT-qPCR. Coronavirus, enterovirus, bocavirus and rhinovirus were found by multiplex RT-qPCR as well as by HTS analyses.

**MAIN CONCLUSIONS:**

Wide virus diversity was found by different methodologies and high frequency of rhinovirus occurrence was confirmed in population in winter, showing its relevance for public health.

Worldwide influenza surveillance was initiated in 1947 to monitor circulating viruses and provides information to support the vaccine-type recommendations by the World Health Organization (Geneva, Switzerland) and the Centers for Disease Control and Prevention (Atlanta, USA). Central laboratories (Lacens) in Brazil are responsible for the occurrence reports about the etiologic agents, types and subtypes of circulating respiratory viruses in patients with influenza-like syndrome (ILS) and severe acute respiratory syndrome (SARS).^1^

The main technique for detecting respiratory viruses in Lacens is reverse transcription-quantitative polymerase chain reaction (RT-qPCR), which advantages are sensitivity, specificity, automation, relatively low cost and so on. However, this technique as well as the conventional RT-PCR are pathogen-specific, which only find pre-defined targets. Thus, a peculiar etiological agent or new genetic variants may not be detected, despite the use of a wide range of sensitive diagnostic tests.^2^

In this context, high-throughput sequencing (HTS) technologies without requiring any prior genomic information provide large volume of nucleotide sequences, resulting in elucidation of unidentified viruses (which were not detectable by the specific detection methods) and furthermore, allow the discovery of new viruses or new genomic variants using metagenomic approaches.

The human population is exposed to increasing burden of infectious diseases caused by the emergence of viruses not yet or rarely characterised. Globalisation, climate change, settlements near wild animal habitats, and increasing numbers of immunocompromised people are likely to contribute to the emergence and spread of new infections.^3^ HTS methods have allowed genomic analysis which is sensitive for unknown or peculiar viruses over previous methodologies, such as the analysis of viral genomes used in the identification or discovery of human herpesvirus 8,^4^ bocavirus,^5^ human parvovirus 4,^6^ human GB virus,^7^ Torque Teno virus,^8^ WU polyomavirus^9^ and KI polyomavirus.^10^

HTS has been used, for example, in the discovery of an arenavirus in three patients who died of a febrile illness a few weeks after transplant of solid organs from a single donor.^11^ The Illumina platform allowed identifying hepatitis A virus among cases of acute febrile illnesses that occurred in Brazil which were suspected as yellow fever.^12^ The same platform was used to identify a new influenza virus from swabs samples and assembly of its genome.^13^ It also led to the detection of viral pathogens in nasopharyngeal aspirate samples from patients with acute lower respiratory tract infections, as a new enterovirus, termed enterovirus 109 (EV109) detected in a cohort of Nicaraguan children with viral respiratory disease.^14^

Thereby, this study aimed to detect unidentified viruses by HTS approach in negative samples of nasopharynx/tracheal secretions for diagnostics targeting several viruses collected from June to August 2016 in the Federal District (DF), Brazil.

## MATERIALS AND METHODS

*Detection of standard respiratory viruses* - The nasopharyngeal and tracheal secretion samples received in the laboratory in June, July and August 2016 were tested for the respiratory virus diagnostic panel performed in Lacen-DF (Public health laboratory located in the Federal District, Brazil) by the standard RT-qPCR (for Influenza A, Influenza B, Human respiratory syncytial virus, Human metapneumovirus, Human adenovirus C, Parainfluenza 1, Parainfluenza 2 and Parainfluenza 3). Those that had negative results were separated and stored in freezer -70ºC for subsequent DNA/RNA extraction for HTS.

*RNA/DNA extraction* - Fifty one samples from June, 55 from July and 39 from August of 2016 were mixed, respectively (one for each month), and treated as three pooled samples. Individual sample had a volume of approximately 0.5 mL (total of 20-25 mL of each pooled sample). The volume was adjusted to 50 mL with 0.1 M sodium phosphate buffer (pH7.2) with Triton-X 100 at the final concentration of 1%. The solution in tubes were agitated for 1 h in a cold chamber, then, centrifuged at 4,800 × g for 40 min. The supernatant was ultracentrifuged with 20% sucrose cushion at 140,000 × g for 1 h using Ti45 rotor (Optima L-90K Ultracentrifuge, Beckman Coulter, Brea, USA). The pellet was resuspended in 0.1 M sodium phosphate buffer and DNA/RNA was extracted from the resuspended pellet following the instructions of the High Pure Viral Nucleic Acid Kit (Hoffmann-La Roche, Basel, Switzerland), without addition of carrier RNA in the lysis buffer. Three DNA/RNA samples were subjected to HTS. Next, part of all three samples were treated with DNase I (Promega, Madison, USA) in order to decrease the human or bacterial DNA in the sample for viral RNA enrichment. However, due to low amount of RNA after DNase I treatment, all three samples were mixed as one pooled sample for RNA HTS.

*High-throughput sequencing* - Approximately two μg of DNA/RNA and RNA samples in RNAStable tube (Biomatrica, San Diego, USA) were sent for HTS to Macrogen Inc. (Seoul, South Korea). The library of DNA/RNA or RNA samples was constructed using TruSeq Standard total RNA kit and sequenced using Illumina HiSeq 2000 with 3 G scale for each in 100 base pared-end.

*Bioinformatics analysis* - Low quality and adapter sequences of the raw data were trimmed using the Trimommatic tool v.036.^15^ Metagenomic reads of each sample were then uploaded to Kaiju for profiling the reads in taxonomic assessment (http://kaiju.binf.ku.dk).^16^ DNA reads were filtered with BWA v0.7.17^17^ and SAMtools v1.9^18^ against reference hg38 (*Homo sapiens*) obtained from GenBank to remove human DNA sequences from HTS dataset *in silico*. Trimmed reads were assembled into contiguous sequences (‘contigs’) with Velvet v.1.2.1^19^ and SPAdes v.3.9^20^ programs. The tBlastx search (https://blast.ncbi.nlm.nih.gov/ Blast.cgi) against the viral genome package (RefSeq Virus, NCBI, https://www.ncbi.nlm.nih.gov/genome/viruses/) which is implemented in the Geneious R8.1 program (Biomatters, Auckland, New Zealand) was used to identify the contig sequences which were viral origins.

*RT-qPCR using PR21 kit* - A part of findings by HTS analysis was confirmed by means of RT-qPCR. The RNA was extracted from original 145 samples using the Magna Pure LC Total Nucleic Acid Kit (Hoffmann-La Roche, Basel, Switzerland), then a pool of five samples were projected to the multiplex RT-qPCR using the XGen PR21 kit (Biometrix, Curitiba, Brazil) for 21 respiratory pathogens (targeting Influenza A, Influenza B, Influenza A H1N1-swl, Coronavirus NL63, Coronavirus 229E, Coronavirus OC43, Coronavirus HKU1, Parainfluenza 1, Parainfluenza 2, Parainfluenza 3, Parainfluenza 4 , Human metapneumovirus A and B, Human respiratory syncytial virus A and B, Rhinovirus, Enterovirus, Parechovirus, Human adenovirus, Bocavirus and *Mycoplasma pneumoniae*).

*Ethics approval and consent to participate* - This research project was reviewed and approved by ethics committee of the Faculty of Health Sciences (University of Brasília), approval letter No. 3.052.443.

## RESULTS

Kaiju program is used for microbial and viral sequence profiling. For June samples a total of 87,317 (0.87%) out of 10,007,522 reads were classified as microbial sequences including virus; for July samples, 79,050 (0.96%) out of 8,215,186; for August samples 91,485 (1.11%) out of 8,271,632 and for the DNase I-treated RNA sample, 401,596 (61.26%) out of 655,558 reads were classified.

The analyses showed that nasopharyngeal secretion pooled samples of June, July and August of 2016 had similar metagenomic profiles in relation to the components of microorganisms classified found: predominance of viruses [Supplementary data (Figure)], followed by bacteria and other microorganisms, such as Archaeas. However, the higher recovery of viral sequences in DNA/RNA samples was considered as misinterpretation by Kaiju program since the later fine analyses using BLASTn found that many of the human DNA sequences present in the samples were recognised as DNA viruses due to the similarity of some genomic regions. After *in silico* filtration of the reads by human DNA sequences, 77.26% of reads were classified as microbial sequences for June (274,269 out of 354,975), 81.22% for July (242,730 out of 298,846) and 82.50% for August (312,605 out of 378,895). This result showed that the contamination of human DNA was problematic for virus sequence search in these samples. As the human chromosomal and microbial DNA masked the presence of RNA viruses in the total DNA/RNA samples, the amount of DNA was reduced by DNase I treatment and the samples were subjected to the HTS again as one RNA sample.

After this treatment, the microorganism profile by Kaiju program had changed, where 4% of the sample was viruses unlike the DNase I-untreated samples [Supplementary data (Figure)]. It is noted that the main microorganisms found in the RNA sample after DNase I treatment were bacteria (80%) [Supplementary data Figure)]. The main bacterial family identified were *Burkholderiaceae, Streptococcaceae, Neisseriaceae*, *Veillonellaceae*, *Pseudomonadaceae*, *Pasteurellaceae*, *Veillonellaceae* and *Mycoplasmataceae*.

The RNA sample was the one that allowed the greater identification of RNA viruses. Therefore, the fine analyses of virus identification were performed using only the RNA sample, since most respiratory viruses possess RNA genomes. A total of 14,952 reads was classified as virus organisms. The main viruses identified using the Kaiju program were of the families *Picornaviridae*, *Herpesviridae*, *Parvoviridae* and *Coronaviridae*, with the emphasis on rhinoviruses (Table I). The same viral families were also found in the DNA/RNA sample reads after the removal of human DNA sequences *in silico*, however, with less frequency. Only cytomegalovirus sequences were found more in DNA/RNA samples than RNA sample (Table I), probably due to the negative effect on cytomegalovirus DNA genomes by DNase I treatment in preparation of RNA sample.

**TABLE I:** Human virus found in DNA/RNA and RNA sample (treated with DNase I) using Kaiju program

Genetic material	Family	Subfamily	Genus	Specie	Total reads	
					DNA/RNA	RNA
dsDNA	*Herpesviridae*	*Betaherpesvirinae*	*Cytomegalovirus*		387	40
ssDNA	*Parvoviridae*	*Parvovirinae*	*Bocaparvovirus*		6	18
ssRNA	*Coronaviridae*	*Orthocoronavirinae*	*Betacoronavirus*		-	16
ssRNA	*Picornaviridae*	-	*Enterovirus*	*Rhinovirus B*	26	1,087
ssRNA	*Picornaviridae*	*-*	*Enterovirus*	*Rhinovirus A*	19	995
ssRNA	*Picornaviridae*	*-*	*Enterovirus*	*Rhinovirus C*	90	2,721
ssRNA	*Picornaviridae*	-	*Enterovirus*	*Enterovirus J*	-	1

ds: double strand; ss: single strand.

Due to low accuracy of the short-read profiling by Kaiju program, the classification up to genus level was shown in this table, except for the *Enterovirus* genus, which was the one with the most reads founded, allowing the virus species definition.

To identify the contig sequences (1,621 contigs assembled by Velvet and 334 by SPAdes assemblers) of viral origin, tBlastx search plugged-in Geneious was used for the known virus genome sequences recorded in the RefSeq Virus (NCBI).

The analyses identified virtually the same viral families/genera found by the Kaiju program: *Picornaviridae*, *Herpesviridae* and *Parvoviridae* using contigs generated by both SPAdes (Table II) and Velvet assemblers (Table III). Only the *Coronaviridae* family was not identified by tBlastx in Geneious (but identified by Kaiju), probably due to limited sensitivity of *de novo* contig assembly, although we chose the most sensitive ones, Velvet and SPAdes.^21^

**TABLE II:** Classification of human virus found in RNA sample (treated with DNase I) by tBlastx using SPAdes assembler

Genetic material	Family	Genus	Species	Contigs number	Alignment length	Query coverage (%)	Number of reads per contig	E-value
ssRNA	*Picornaviridae*	*Enterovirus*	*Rhinovirus B*	7	93 - 579	46.80 - 98.63	47 - 1,194	2.72 x 10^-142^ - 1.67 x 10^-42^
ssRNA	Picornaviridae	*Enterovirus*	*Rhinovirus A*	12	177 - 1,158	53.96 - 99.48	11 - 254	3.81 x 10^-76^ - 1.99 x 10^-11^
ssRNA	*Picornaviridae*	*Enterovirus*	*Rhinovirus C*	10	225 - 1,239	64.71 - 99.68	4 - 2,470	5.38 x 10^-148^ - 4.37 x 10^-32^
ssRNA	Picornaviridae	Enterovirus	Enterovirus B	1	174	80.93	43	2.30 x 10^-16^
ssRNA	*Picornaviridae*	*Enterovirus*	*Enterovirus C*	1	231	97.88	36	9.96 x 10^-26^

Ss: single strand.

It is observed in Table II (contigs assembled by SPAdes) that the number of contigs varied from 1 (*Enterovirus B* and *C*) to 12 (*Rhinovirus A*), with sizes of alignment between 93 and 1,239 nucleotides. The number of reads per contig also varied greatly, from 4 to 2,470 with the highest amount of reads per contig found in the *Rhinovirus C* species. The e-values from 5.38 x 10^-148^ to 1.99 x 10^-11^ indicates statistical significance in the alignments found using the SPAdes as assembler.

**TABLE III:** Classification of human virus found in RNA sample (treated with DNase I) by tBlastx using Velvet assembler

Genetic material	Family	Genus	Species	Contigs number	Alignment length	Query coverage (%)	Number of reads per contig	E-value
dsDNA	*Herpesviridae*	*Cytomegalovirus*	*Human betaherpesvirus 5*	3	96 - 153	95.05 - 96.84	23 - 24	3.15 x 10^-32^ - 4.76 x 10^-16^
ssDNA	*Parvoviridae*	*Bocaparvovirus*	*Primate bocaparvovirus 1*	4	96 - 99	95.05 - 98.02	8 - 13	2.15 x 10^-18^ - 1.34 x 10^-16^
ssRNA	*Picornaviridae*	*Enterovirus*	*Rhinovirus B*	29	93 - 570	63.00 - 100.00	3 - 668	2.01 x 10^-117^ - 3.20 x 10^-11^
ssRNA	*Picornaviridae*	*Enterovirus*	*Rhinovirus A*	62	93 - 435	53.57 - 100.00	4 - 257	2.65 x 10^-68^ - 6.05 x 10^-11^
ssRNA	*Picornaviridae*	*Enterovirus*	*Rhinovirus C*	54	87 - 885	64.15 - 100.00	11 - 962	1.23 x 10^-152^ - 7.86 x 10^-11^
ssRNA	*Picornaviridae*	*Enterovirus*	*Enterovirus B*	1	174	80.93	43	2.30 x 10^-16^
ssRNA	*Picornaviridae*	*Enterovirus*	*Enterovirus C*	1	117	77.48	72	1.05 x 10^-13^
ssRNA	*Picornaviridae*	*Enterovirus*	*Enterovirus E*	1	99	98.02	9	2.33 x 10^-11^

ss: single strand; ds: double strand.

Table III shows the tBlastx results of contigs assembled by Velvet. The number of contigs varied between 1 (*Enterovirus B*, *C* and *E*) and 62 (*Rhinovirus A*) with sizes of alignment between 87 and 885 nucleotides. The number of reads per contig ranged from 3 to 962, with the highest number of reads found also for *Rhinovirus C*. E-values from 1.23 x 10^-152^ to 2.33 x 10^-11^ were also low, showing statistical significance of the alignment. The comparison of Tables II and III reveals that, when using Velvet as assembler, more contigs were formed, although with smaller length and fewer reads per contig. But this is important to identify more viruses in less abundance, as *Human betaherpesvirus 5*. Human bocavirus and *Enterovirus E* have been identified using Velvet, but not when using SPAdes as assembler.

The Rhinovirus sequences showed greater abundance by the presence of reads in the sample, especially *Rhinovirus C*, that presented highest reads number (observed in Kaiju analysis), and the highest reads number per contig (in tBlastx analysis). This shows the relevance of this one virus in these negative samples by the standard RT-qPCR.

The presence of respiratory viruses in the samples was confirmed by the PR21 multiplex RT-qPCR kit, which identifies 21 respiratory pathogens. Coronavirus 63 (CT = 30), coronavirus 43 (CT = 22-31), coronavirus HKU 1 (CT = 20-33), enterovirus (CT = 33), bocavirus (CT = 19-35) and mainly rhinovirus (CT = 21-36) were found (Figure). The RT-qPCR assay showed the presence of coronaviruses which detection was failed by the HTS Blast analyses.

**Figure:**
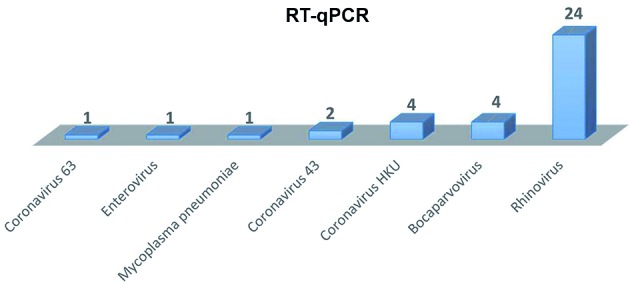
Viruses found by PR21 multiplex reverse transcription-quantitative polymerase chain reaction (RT-qPCR) in nasopharyngeal and tracheal secretion samples which were negative by the standard RT-qPCR of influenza-like surveillance on June, July and August collected in Brasília, 2016.

## DISCUSSION

The HTS has been successfully applied in several fields of virology, including virus discovery, complete virus genome sequence determination, and genomic variant analyses.^22^

Lacen-DF performs diagnosis of the respiratory virus by the standard RT-qPCR protocol with restricted targets, thus, many viruses may not be identified. The samples referred to Lacen are from patients of public and private hospitals with respiratory virus infection suspected, whether hospitalised or not. Approximately 40% of the samples forwarded are negative for the virus detection.

In this study, *Human betaherpesvirus 5*, *Primate bocaparvovirus 1*, *Betacoronavirus*, *Enterovirus* and three species of rhinovirus were found by at least two different types of analyses in samples of patients from the DF, Brazil, with flu symptoms. *Human betaherpesvirus 5* (Cytomegalovirus) was identified by analyses with Kaiju and Geneious program, but not with PR21 RT-qPCR because specific primers for this pathogen were not included in the kit. DNA viruses as herpesvirus and parvovirus were also found in the sample treated with DNase I. We assume that DNase I treatment doesn’t exclude totally DNA in the sample, thus, herpesvirus and parvovirus reads were recovered. Coronavirus was identified with the Kaiju program but not by the contig analyses by tBlastx. However, its presence was confirmed by RT-qPCR of PR21 kit. Bocavirus (or *Primate bocaparvovirus 1*), and *Enterovirus* was also identified by PR21 RT-qPCR, Kaiju and Geneious programs only using Velvet, which is more sensitive method than SPAdes for contig assembly. *Mycoplasma pneumoniae* was found by PR21 RT-qPCR and many bacteria of the genus mycoplasma were also identified using the Kaiju program. Rhinovirus was identified using all methods used is this study.

These variations in results are explained by the differences in specificity and sensitivity in each method. For some cases, the limited sensitivity of HTS is explained by mixing some clinical samples forming one pooled sample for the cost reduction. Therefore, this sample preparation may mask the presence of viruses in low incidence or concentration. The limited capacity of *de novo* assembly programs is still a main problem in metagenomic analyses. Velvet and SPAdes *de novo* assemblers were used to build contigs in this study since these were reported as sensitive contig assembler for viral metagenomic study,^21^ although there was no best *de novo* assembler concluded by the same authors. HTS was reported to be less sensitive than RT-qPCR for some respiratory virus detection in such reasons.^23^

Rhinovirus was the major viruses found in patients in the DF, Brazil with ILS or SARS samples which were negative by standard RT-qPCR of influenza-like surveillance (targeting Influenza A and B, Respiratory Syncytial Virus, Human Metapneumovirus, Adenovirus, Parainfluenza 1, 2 and 3). Several studies have shown the importance of rhinoviruses in acute respiratory infection in Brazil, especially in children.^24^ ^,^ ^25^ ^,^ ^26^ A study involving 120 children less than 12 years old in São Paulo, Brazil, found that human rhinoviruses (HRV) of species A, B, and C were the most frequent agents of acute respiratory infections (ARI). Moreover, these agents are also associated with up to 70% of virus-related wheezing exacerbations. The most recently identified *Rhinovirus C* has been detected in association with bronchiolitis, wheezing, and asthma exacerbations requiring hospitalisation.^27^

Other study made with hospitalised patients at an academic care centre in Southern Brazil shows that HRV was usually detected in hospitalised children with respiratory infections and was often present in viral co-detections, mainly with enterovirus and respiratory syncytial virus. Comorbidities are closely associated with HRV infections, and this virus predominates during colder seasons,^28^ period of which the samples used in this work were collected (winter). In investigations around the world, HRV, as well as agents causing acute respiratory infections, has its peak activity associated with the colder periods of the year.^29^ ^,^ ^30^

In this study, the relevance of HRV in public health in Brazil was again recognised and we suggest the inclusion of HRV for influenza-like surveillance.

## References

[1] Ministry of Health (2016). Guide to the influenza surveillance laboratory network in
Brazil. Ministry of Health.

[2] Juvén T, Mertsola J, Waris M, Leinonen M, Meurman O, Roivainen M (2000). Etiology of community-acquired pneumonia in 254 hospitalized
children. Pediatr Infect Dis J.

[3] Morens DM, Folkers GK, Fauci AS (2004). The challenge of emerging and re-emerging infectious
diseases. Nature.

[4] Chang Y, Cesarman E, Pessin MS, Lee F, Culpepper J, Knowles DM (1994). Identification of herpesvirus-like DNA sequences in
AIDS-associated Kaposi's sarcoma. Science.

[5] Allander T, Tammi MT, Eriksson M, Bjerkner A, Tiveljung-Lindell A, Andersson B (2005). Cloning of a human parvovirus by molecular screening of
respiratory tract samples. Proc Natl Acad Sci USA.

[6] Jones MS, Kapoor A, Lukashov VV, Simmonds P, Hecht F, Delwart E (2005). New DNA viruses identified in patients with acute viral infection
syndrome. J Virol.

[7] Simons JN, Pilot-Matias TJ, Leary TP, Dawson GJ, Desai SM, Schlauder GG (1995). Identification of two flavivirus like genomes in the GB hepatitis
agent. Proc Natl Acad Sci USA.

[8] Nishizawa T, Okamoto H, Konishi K, Yoshizawa H, Miyakawa Y, Mayumi M (1997). A novel DNA virus (TTV) associated with elevated transaminase
levels in post transfusion hepatitis of unknown etiology. Biochem Biophys Res Commun.

[9] Gaynor AM, Nissen MD, Whiley DM, Mackay IM, Lambert SB, Wu G (2007). Identification of a novel polyomavirus from patients with acute
respiratory tract infections. PLoS Pathog.

[10] Allander T, Andreasson K, Gupta S, Bjerkner A, Bogdanovic G, Persson MA (2007). Identification of a third human polyomavirus. J Virol.

[11] Palacios G, Druce J, Du L, Tran T, Birch C, Briese T (2008). A new arenavirus in a cluster of fatal transplant-associated
diseases. N Engl J Med.

[12] Conteville LC, de Filippis AMB, Nogueira RMR, de Mendonça MCL, Vicente ACP (2018). Metagenomic analysis reveals Hepatitis A virus in suspected
yellow fever cases in Brazil. Mem Inst Oswaldo Cruz.

[13] Yongfeng H, Fan Y, Jie D, Jian Y, Ting Z, Lilian S (2011). Direct pathogen detection from swab samples using a new
high-throughput sequencing technology. Clin Microbiol Infect.

[14] Yozwiak NL, Skewes-Cox P, Gordon A, Saborio S, Kuan G, Balmaseda A (2010). Human enterovirus 109 a novel interspecies recombinant
enterovirus isolated from a case of acute pediatric respiratory illness in
Nicaragua. J Virol.

[15] Bolger AM, Lohse M, Usadel B (2014). Trimmomatic a flexible trimmer for Illumina sequence
data. Bioinformatics.

[16] Menzel P, Ng KL, Krogh A (2016). Fast and sensitive taxonomic classification for metagenomics with
Kaiju. Nat Commun.

[17] Li H, Durbin R (2009). Fast and accurate short read alignment with Burrows-Wheeler
transform. Bioinformatics.

[18] Li H, Handsaker B, Wysoker A, Fennell T, Ruan J, Homer N (2009). The sequence alignment/map format and SAMtools. Bioinformatics.

[19] Zerbino DR, Birney E (2008). Velvet algorithms for de novo short read assembly using de Bruijn
graphs. Genome Res.

[20] Bankevich A, Nurk S, Antipov D, Gurevich AA, Dvorkin M, Kulikov AS (2012). SPAdes a new genome assembly algorithm and its applications to
single-cell sequencing. J Comput Biol.

[21] Blawid R, Silva JMF, Nagata T (2017). Discovering and sequencing new plant viral genomes by
next-generation sequencing description of a practical
pipeline. Ann Appl Biol.

[22] Capobianchi MR, Giombini E, Rozera G (2013). Next-generation sequencing technology in clinical
virology. Clin Microbiol Infect.

[23] Thorburn F, Bennett S, Modha S, Murdoch D, Gunson R, Murcia PR (2015). The use of next generation sequencing in the diagnosis and typing
of respiratory infections. J Clin Virol.

[24] Arruda E, Hayden FG, McAuliffe JF, Sota MA, Mota SB, McAuliffe MI (1991). Acute respiratory viral infections in ambulatory children in
urban northeast Brazil. J Infect Dis.

[25] Costa LF, Yokosawa J, Mantese OC, Oliveira TFM, Silveira HL, Nepomuceno LL (2006). Respiratory viruses in children younger than five years old with
acute respiratory disease from 2001 to 2004 in Uberlândia, MG,
Brazil. Mem Inst Oswaldo Cruz.

[26] Fawkner-Corbett DW, Khoo SK, Duarte MC, Bezerra P, Bochkov YA, Gern JE (2015). Rhinovirus-C detection in children presenting with acute
respiratory infection to hospital in Brazil. J Med Virol.

[27] Moreira LP, Kamikawa J, Watanabe AS, Carraro E, Leal E, Arruda E (2011). Frequency of human rhinovirus species in outpatient children with
acute respiratory infections at primary care level in Brazil. Pediatr Infect Dis J.

[28] Leotte J, Trombetta H, Faggion HZ, Almeida BM, Nogueira MB, Vidal LR (2017). Impact and seasonality of human rhinovirus infection in
hospitalized patients for two consecutive years. J Pediatr.

[29] Huei-Min H, Shu-Li Y, Chih-Jung C, Cheng-Hsun C, Chen-Yen K, Kuan-Ying AH (2019). Molecular epidemiology and clinical features of rhinovirus
infections among hospitalized patients in a medical center in
Taiwan. J Microbiol Immunol Infect.

[30] Bellei N, Carraro E, Perosa A, Watanabe A, Arruda E, Granato C (2008). Acute respiratory infections and influenza-like illness viral
etiologies in Brazilian adults. J Med Virol.

