# Supplementary material for: Human virome in nasopharynx and tracheal secretion samples
Source: Mem Inst Oswaldo Cruz. 2019 Oct 3;114:e190198. doi: 10.1590/0074-02760190198 (PMC6779266; doi:10.1590/0074-02760190198)
Supplement: Supplementary file 1 [file 1678-8060-mioc-114-e190198-s.pdf]

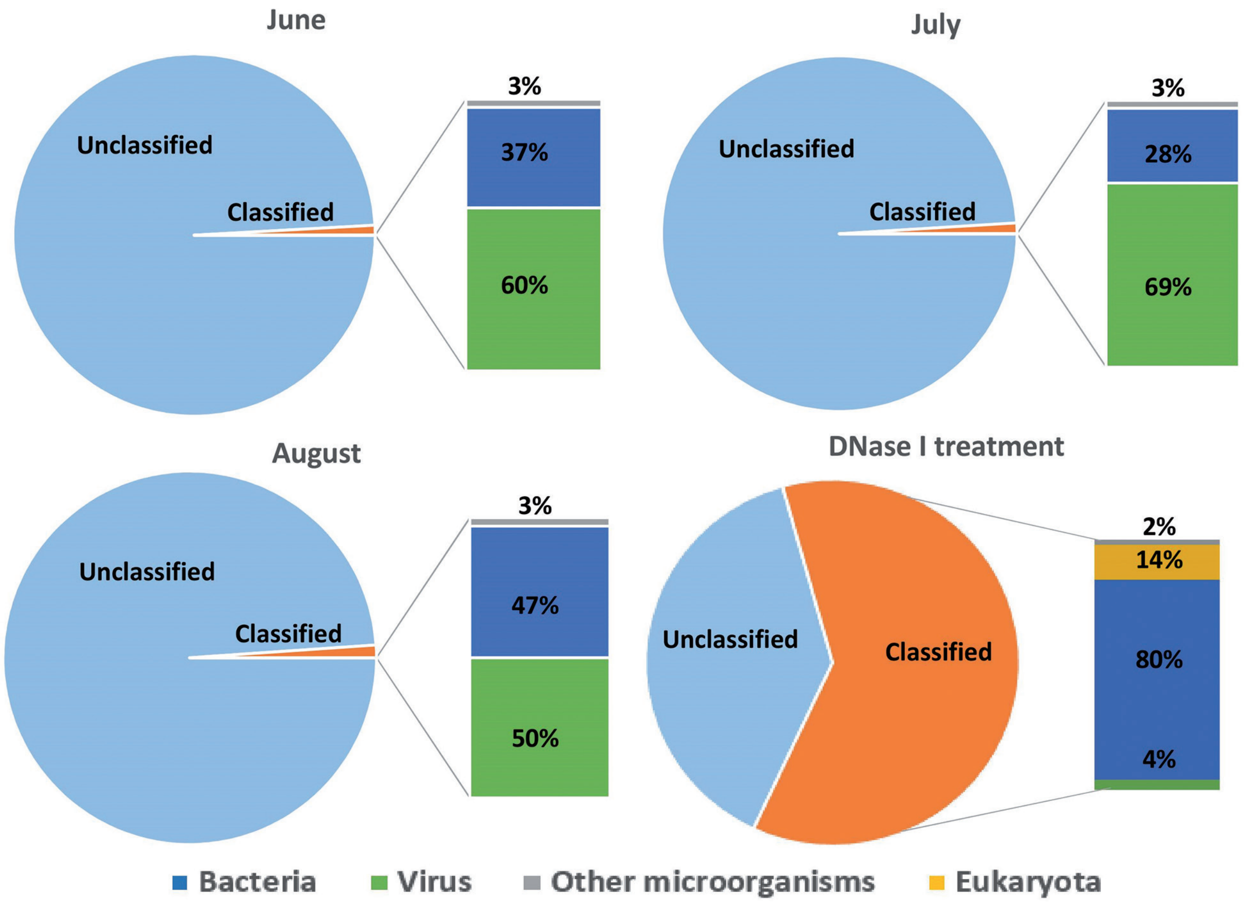

Microorganism classified profiles found by Kaiju program in the DNA/RNA preparations of the nasopharyngeal and tracheal secretion samples of June, July and August (2016) and the RNA preparation treated with DNase I.

CC BY
